# Supplementary figures and images for: Chronic stress from adolescence to adulthood increases adiposity and anxiety in rats with decreased expression of Krtcap3
Source: Front Genet. 2024 Jan 23;14:1247232. doi: 10.3389/fgene.2023.1247232 (PMC10844407; doi:10.3389/fgene.2023.1247232)

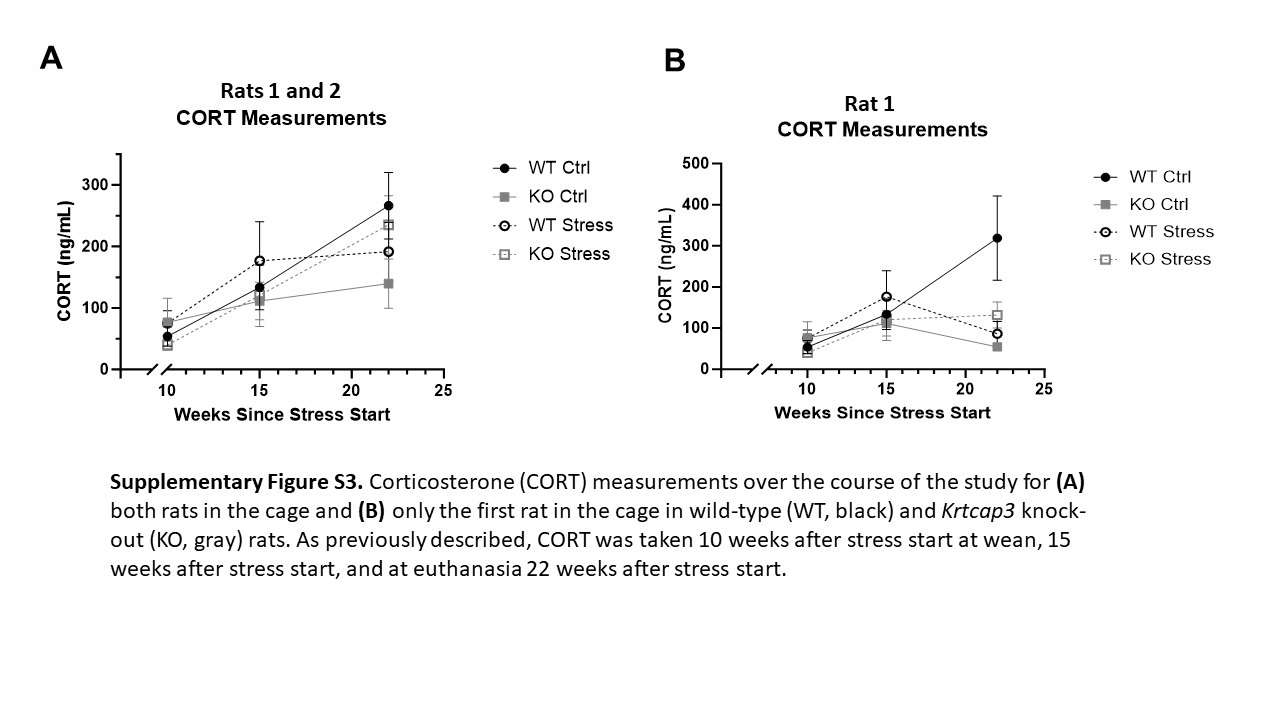

Supplement: Supplementary file 1 [file Image3.tif]

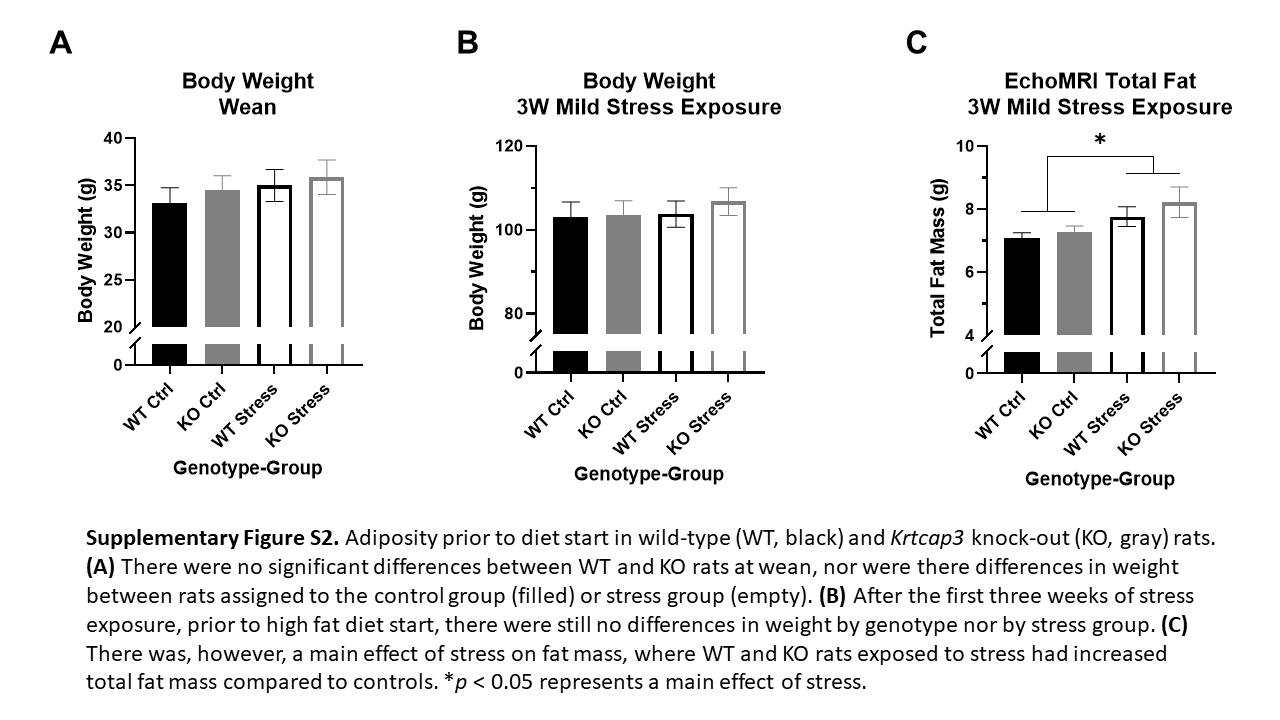

Supplement: Supplementary file 2 [file Image2.tif]

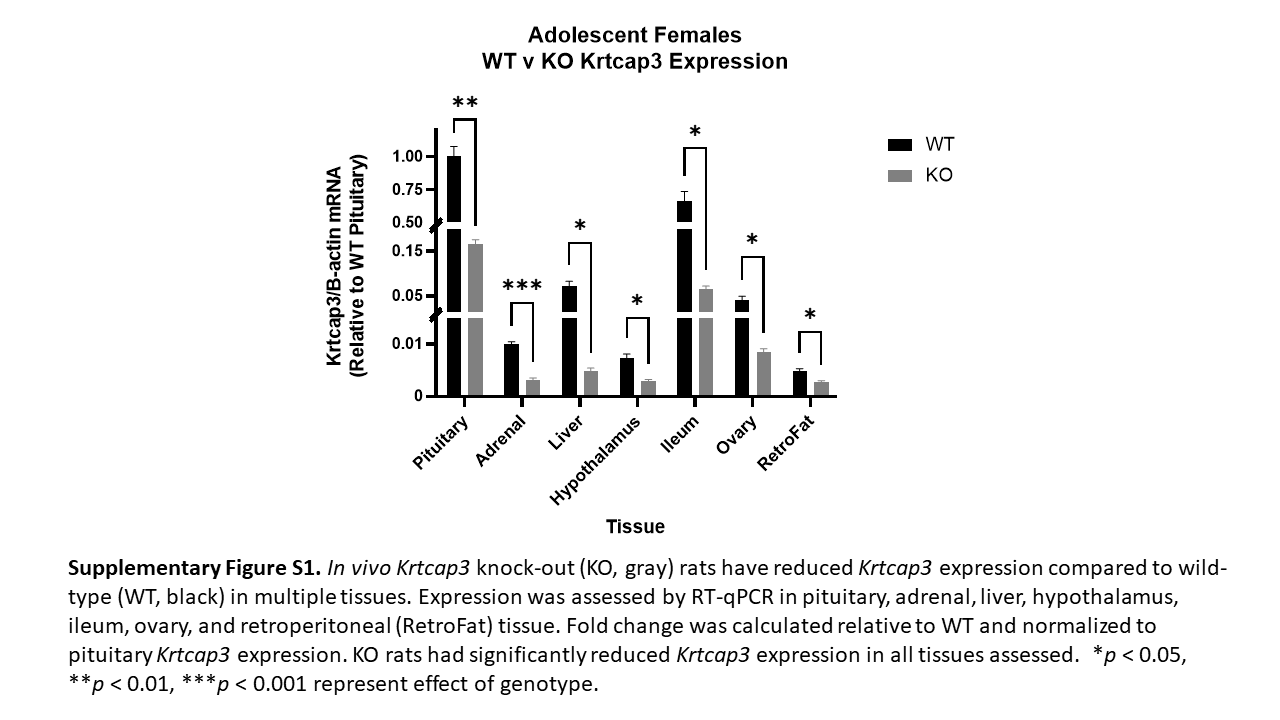

Supplement: Supplementary file 3 [file Image1.tif]
